# Transcription Factor-7-Like-2 (TCF7L2) in Atherosclerosis: A Potential Biomarker and Therapeutic Target

**DOI:** 10.3389/fcvm.2021.701279

**Published:** 2021-09-09

**Authors:** Junyi Li, Li Zhou, Xinping Ouyang, Pingping He

**Affiliations:** ^1^School of Nursing, Hengyang Medical College, University of South China, Hengyang, China; ^2^Department of Pathology, Chongqing Public Health Medical Center, Southwest University Public Health Hospital, Chongqing, China; ^3^Hengyang Key Laboratory of Neurodegeneration and Cognitive Impairment, Department of Physiology, Hunan Province Cooperative Innovation Center for Molecular Target New Drug Study, Hengyang Medical College, The Neuroscience Institute, University of South China, Hengyang, China

**Keywords:** TCF7L2, rs7903146, metabolism, atherosclerosis, CVD

## Abstract

Transcription factor-7-like-2 (TCF7L2), a vital member of the T-cell factor/lymphoid enhancer factor (TCF/LEF) family, plays an important role in normal human physiological and pathological processes. TCF7L2 exhibits multiple anti-atherosclerotic effects through the activation of specific molecular mechanisms, including regulation of metabolic homeostasis, macrophage polarization, and neointimal hyperplasia. A single-nucleotide substitution of TCF7L2, rs7903146, is a genetic high-risk factor for type 2 diabetes and indicates susceptibility to cardiovascular disease as a link between metabolic disorders and atherosclerosis. In this review, we summarize the anti-atherosclerosis effect and novel mechanisms underlying the function of TCF7L2 to elucidate its potential as an anti-atherosclerosis biomarker and provide a novel therapeutic target for cardiovascular diseases.

## Introduction

Atherosclerosis precedes and forecasts the pathological process of cardiovascular disease (CVD), the leading cause of mortality worldwide (1, 2). Atherosclerosis is characterized by complex pathological progression involving endothelial damage, local chronic inflammation, and metabolic disorders (3). The onset of atherosclerosis in endothelium stimulates chemokine releases and recruits circulating monocytes, which then differentiate into M1 macrophages in the local inflammatory environment (4). Additionally, subendothelial M1 macrophages not only secrete proinflammatory cytokines but also transform into foam cells through excessive lipid uptake. However, foam cells are prone to apoptosis or necrosis owing to cytotoxicity, and the debris and lipids from foam cells inflate the necrotic core of atherosclerotic plaques. Vascular smooth muscle cell (VSMC) migration is another pathologic feature of atherogenesis, which thickens the vascular walls and narrows the vascular lumen. Even worse, once these atherosclerotic plaques rupture and the necrotic core breaks through into the vascular lumen, patients will suffer from acute CVD events, such as stroke or myocardial infarction. In addition to the local microenvironment, atherogenesis is affected by multiple systemic factors such as obesity, dyslipidemia, and insulin resistance (IR). Recently, metabolic syndrome, the simultaneous presence of various risk factors related to metabolism, has been identified as a prominent risk factor for atherosclerosis (5).

TCF7L2 is well characterized as a member of the T-cell factor/lymphoid enhancer factor (TCF/LEF) family, whose remaining members are LEF1, TCF7, and TCF7L1 in humans. It was also previously called T-cell factor 4 (TCF4), not to be mistaken for transcription factor 4 (6). Korinek et al. (7) discovered TCF7L2 *via* the hybridization screening of *TCF7* cDNA for the first time and described TCF7L2 as an important transcription factor of Wnt signaling. During embryogenesis, TCF7L2 promotes embryonic organ development as a key player in Wnt signaling (7, 8). In adults, TCF7L2 has attracted widespread attention because of its significant genetic correlation with the high risk of type 2 diabetes (T2D), which alters the function and survival of pancreatic β cells (9–13). Currently, TCF7L2 has drawn increasing academic attention because it has been reported to be associated with inflammation, metabolism, and atherosclerosis (14, 15). Studies have shown that silencing TCF7L2 leads to insufficient insulin secretion, impaired adipogenic differentiation, blood-vessel dysplasia, and lipid accumulation (16–18). In contrast, overexpression of TCF7L2 promotes macrophage polarization (M2) and inhibits neointimal hyperplasia (19, 20). This evidence indicates the anti-atherosclerotic effects of TCF7L2. With an in-depth study of genomics, single-nucleotide polymorphisms (SNPs) have been found to be related to the genetic susceptibility to atherosclerosis and the distribution of high-risk populations; of these, TC7FL2 rs7903146 is one of the most notable SNPs (21–23). These findings indicate that TCF7L2 plays a vital role in anti-atherosclerosis and can be considered as a potential biomarker for the treatment of CVD. Therefore, in this review, we summarize the structure, function, and anti-atherosclerosis effects of TCF7L2 in order to provide insight for the development of an alternative treatment strategy for CVD.

## Structural and Functional Characteristics of TCF7L2

In humans, TCF7L2 is located on chromosome 10q25.2-q25.3; it spans over 217,432 bp of DNA and consists of 17 exons. The full-length TCF7L2 protein contains 596 amino-acid residues in a certain subsequence of the N terminal, β catenin-binding domain, Groucho-binding sequence, HMG box-DNA-binding domain (HGM-DBD), cysteine clamp (C clamp), and C terminal (24, 25). With the help of HGM-DBD, TCF7L2 can recognize specific DNA subsequences (5′-xCTTTGATx-3′) in the double-helix dimple and trigger transcription factor activity (17, 26). Until now, the C clamp has been considered to assist the binding of HGM-DBD with certain DNA sequences, although the C clamp contains an alternative DNA-binding domain (5′-xTGCCGCx-3′) without transcription regulatory activity (27). Remarkably, TCF7L2 exerts dual transcription regulatory effects on target genes influenced by the transcriptional co-activator β-catenin or transcriptional co-repressor transducin-like enhancer of split (TLE)/Groucho (28–30). With Wnt signaling stimulation, increased amounts of β-catenin are imported into the nucleus, where they subsequently assemble into the β-catenin/TCF7L2 complex. In addition, β-catenin functions as a scaffold to assist the binding of the β-catenin/TCF7L2 complex to the promoter of target genes and thus enhance promoter activity. In the absence of Wnt/β-catenin signaling, the co-repressor TLEs preferentially occupy TCF7L2 by the glutamine-rich (Q) domain and recruit histone methyltransferases or histone deacetylases to silence downstream genes (31, 32). Taken together, TCF7L2 contains two DNA-binding domains (HGM-DBD and C clamp), but only HGM-DBD can activate transcription. Furthermore, TCF7L2 is subject to dual regulation by the transcriptional co-activator β-catenin or transcriptional co-repressor TLE/Groucho.

## TCF7L2 and Wnt Signaling

Wnt signaling plays a protective role in the development of atherosclerotic CVDs. Many studies have shown that Wnt signaling prevents the development of atherosclerosis *via* a series of processes, including glucolipid metabolism, macrophage polarization, and neointimal hyperplasia (33–36). Upon activation of canonical Wnt signaling, accumulated β-catenin binds to TCF7L2 in the nucleus and regulates the expression of downstream genes. This signaling pathway is widely present in pancreatic islets and adipocytes, where it balances glucose and lipid metabolism. TCF7L2 is the terminal executor of the Wnt signaling pathway, which directly acts on the promoters of downstream genes. Insulin and glucagon exert opposite effects on blood glucose levels; glucagon-like peptide 1 (GLP-1) promotes insulin secretion, while inhibiting glucagon secretion in a blood glucose concentration-dependent manner. Yi et al. (37) found that the promoters of both GLP-1 and glucagon contain TCF7L2-binding sites. However, TCF7L2 upregulates GLP-1 expression in gut endocrine cells but downregulates glucagon expression in pancreatic α cells, exerting powerful effects on insulin synthesis and balance of blood glucose levels (37). Additionally, macrophages have been proven to be a source and effector of Wnt signaling, and Wnt signaling greatly affects macrophage phenotypes. Wnt signaling can induce myeloblasts to differentiate into monocytes in the bone marrow (38) and can promote macrophage polarization into M2 phenotypes in the peripheral residences, with a pattern of decreasing expression of TNF-α and IL-6 and increasing expression of the M2 marker CD206 (39–41). Taken together, the Wnt signaling pathway has been proven to balance blood glucose levels and alleviate local inflammation, which is defined as an anti-atherosclerosis action. Considering TCF7L2 to be a determinant element of Wnt signaling, we will further explore the role of TCF7L2 in metabolism, inflammation, and atherosclerosis.

## TCF7L2 rs7903146 As a Risk Predictor Of CVD

With the advances in genome sequencing, the identification of gene polymorphisms in populations has been shown to be important in disease risk prediction (42). SNPs comprise a type of gene polymorphisms and specifically refer to single-nucleotide substitutions of one base for another that have more than 1% incidence in the general population (43). Many gene polymorphisms have been confirmed to be involved in the development of atherosclerosis, such as rs7903146 in TCF7L2.

**Figure 1:**
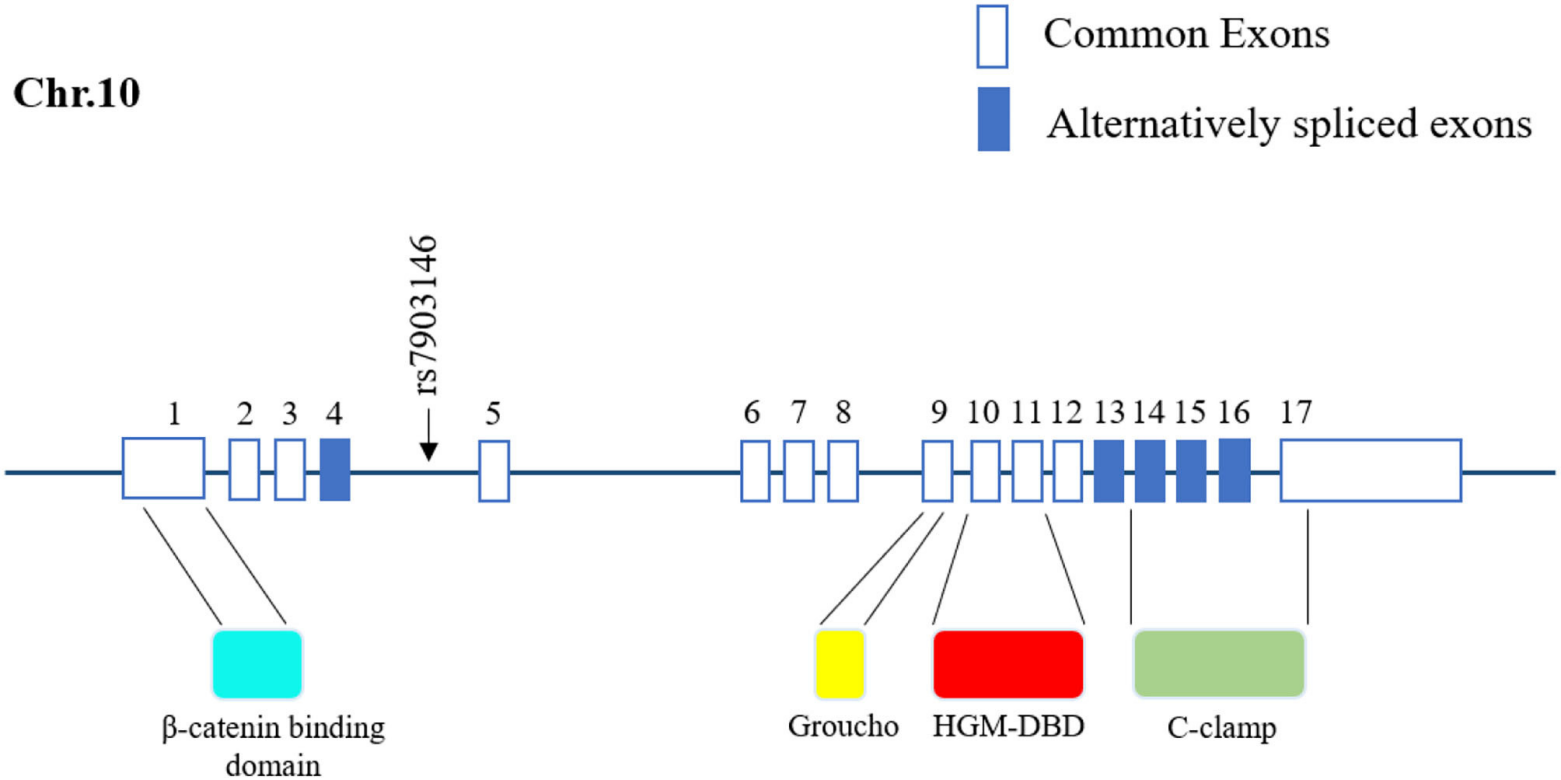
Location of rs7903146 in human *TCF7L2*. Representative 17 exons and location of rs7903146 on human *TCF7L2*. Alternatively spliced exons are colored, and the black arrow represents alternative transcription start sites. rs7903146 is located in exon 4 of TCF7L2. TCF7L2 comprises several domains, including the β catenin-binding domain, Groucho-binding sequence, HMG box-DNA-binding domain (HGM-DBD), and cysteine clamp (C-clamp).

SNPs can be classified into two categories according to their distribution on DNA: linked SNPs and causative SNPs (44, 45). Linked SNPs do not reside within genes and do not affect protein production or function. Causative SNPs are located within the coding region and regulatory subsequence and can thus influence gene transcription or translation. Among causative SNPs, rs7903146, located in exon 4 of TCF7L2, is well known for conferring a genetic predisposition to T2D (Figure 1) (9, 46). rs7903146 influences the activity of the TCF7L2 promoter and alters the alternative splicing of exon 4 (47–50). The T-allele genotype of TCF7L2 shows a higher degree of open chromatin, and the promoter is accessible to transcription factors. Additionally, rs7903146 is regarded as a cis-regulatory variation wherein the T-allele genotype exhibits robust enhancer activity to promote TCF7L2 transcription (49, 50). Conversely, TCF7L2 protein levels are decreased in T-allele carriers, in contrast to elevated TCF7L2 mRNA levels (11, 51). A possible hypothesis for this inconsistency is that rs7903146 reduces exon 4 cutting from TCF7L2 transcripts (47, 48). Notably, this *TCF7L2* transcript containing exon 4 exhibits high mRNA but gets rarely encoded into mature protein, which caused the discrepancy in transcription and translation. In addition, the expression of this *TCF7L2* splice variant increases endoplasmic reticulum stress, triggers the ER-associated degradation pathway to degrade, and ultimately induces irreversible apoptosis (48, 52, 53). Thus, rs7903146 in TCF7L2 not only affects the expression of TCF7L2 but also causes cell dysfunction.

Atherosclerosis is well documented to be the primary pathological basis of CVD and results from subendothelial lipid accumulation and local chronic inflammation. rs7903146 induces postprandial dyslipidemia, causing high triglyceride (TG), high low-density lipoprotein (LDL)-C, and low high-density lipoprotein (HDL)-C levels (54, 55). Some evidence implies that Apo-B is overproduced, but ApoA-I is lacking in T-allele carriers with a high-fat diet (54). ApoA-I is well documented to be a beneficial apolipoprotein in the cardiovascular system (56, 57); ApoA-I accepts intracellular cholesterol efflux from the membrane transporter ABCA1, assembles it into HDL, and transports the excess cholesterol to the liver for metabolism. Deficiency of ApoA-I reduces the feedback regulation of the cardiovascular system in response to dyslipidemia and increases subendothelial lipid accumulation (21, 58). Additionally, TCF7L2 rs7903146 aggravates pancreatic β-cell dysfunction and IR and thus presents as a metabolic correlation in CVD and T2D. Islet morphology analysis showed a 20% decrease in β-cell mass and a 30% increase in α-cell mass within islets from T/T genotype carriers (59). As for islet function, T/T-allele homozygotes exhibited 3.2 mg/dl higher baseline fasting glucose levels than C/C allele homozygotes. In addition, TCF7L2 rs7903146 reduces the efficiency of glipizide and metformin in pre-diabetic or newly diagnosed T2D subjects (60, 61). As previously mentioned, TCF7L2 can promote GLP-1 expression and sensitize pancreatic β-cells to blood glucose, but TCF7L2 rs7903146 causes a decrease in protein levels. These underlying mechanisms are consistent with the function and pathological changes in the islets of T/T allele homozygotes.

In summary, TCF7L2 rs7903146 has a great impact on metabolic balance, linking dyslipidemia and hyperglycemia, and results in greater risk of CVD in T-allele carriers, especially in T2D patients (21). Decades ago, Bielinski et al. (62) found that rs7903146 slightly increased the CVD risk in patients with early-onset diabetes, and they attributed this risk to diabetes without further research. However, this explanation was not all-inclusive, because atherosclerosis was regarded as a multi-cause disease. On the one hand, TCF7L2 rs7903146 induces the development of T2D by damaging pancreatic β-cell function and stimulating IR, which causes the vascular endothelium to be more accessible to atherosclerosis (18, 63). On the other hand, rs7903146 can lead to dyslipidemia and arterial intimal hyperplasia, both of which causes irreversible damage to the cardiovascular system (54, 64). Above all, TCF7L2 rs7903146 appears to have an intricate relationship with atherosclerosis. Although T2D is an indispensable factor, dyslipidemia and other factors are also involved in this relationship. Therefore, TCF7L2 rs7903146 can be regarded as a genetic risk factor for CVD, which could be useful to guide clinical treatment.

## Anti-atherosclerotic Role of TCF7L2 In Metabolic Balance

**Figure 2:**
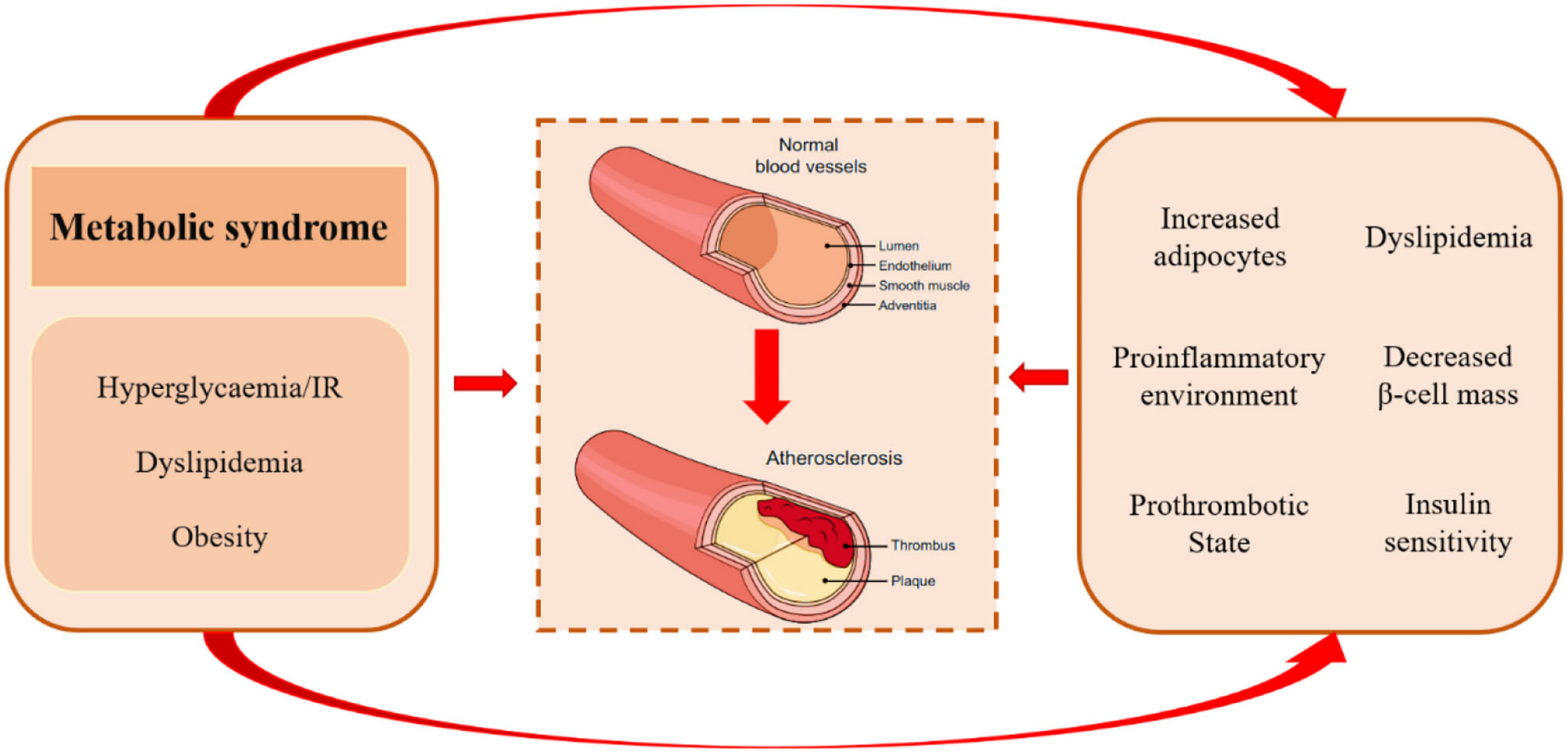
Pathological mechanisms of atherosclerosis in metabolic syndrome. Metabolic syndrome comprises a complex group of metabolic disorders characterized by hyperglycemia/insulin resistance, dyslipidemia, and obesity. It may accelerate atherogenic progression *via* increased adipocytes, proinflammatory environment, prothrombotic state, excess concentration of free fatty acids, unfavorable lipidomics, and decreased insulin levels and insulin sensitivity.

As mentioned previously, TCF7L2 rs7903146 is a genetic risk factor for CVD, which cause a reduction in TCF7L2 protein. However, TCF7L2 is a functional transcription factor with pleiotropic anti-atherosclerotic effects on glucose and lipid metabolism. Metabolic syndrome comprises a complex group of pathological circumstances associated with metabolic and proinflammatory states, which play an important role in the atherosclerotic process by gathering atherogenic risk factors (Figure 2) (65). It is noteworthy that TCF7L2 is widely distributed in pancreatic islets, adipose tissue, and the liver and regulates their function and metabolism.

### TCF7L2 Improves Pancreatic β-Cell Function and Insulin Sensitivity

A large sample investigation involving 584 participants revealed that compared to non-T2D participants, T2D patients suffer from more severe carotid atherosclerotic stenosis, appearing as larger lipid necrosis cores with increasing calcification (66). T2D patients are extremely susceptible to CVD, which has become the most common cause of death in T2D (67). Similar to other metabolic diseases, T2D shares several pathological factors with atherosclerosis. Insulin insufficiency, the chief culprit in T2D, also causes damage to vessel walls and increases inflammasome activity (68–70). All these pathologies propel the progression of atherosclerosis. TCF7L2 mutations or deficiency have been shown to impair both pancreatic β-cell and insulin function in *in vivo* and *in vitro* experiments (18, 71, 72).

As the exclusive source of insulin, β cells are vital for maintaining metabolic balance and organ functions. On the one hand, TCF7L2 is positively related to β-cell mass (16, 59). As quiescent cells, β cells have a long-life span with low proliferation ability, but their regeneration can be affected by tissue damage and increasing demands (73–75). In these situations, TCF7L2 is required for β-cell renewal and regeneration through JAK2/STAT3/Ngn3 signaling. In addition, TCF7L2 directly reduces mitochondrial permeability and inhibits β-cell apoptosis *via* the GSK-3β/p53-dependent pathway (76–78). On the other hand, TCF7L2 affects the most important β-cell function, insulin secretion, from two aspects: proinsulin maturation and Ca^2+^ voltage-gated channel (CAV) activity. To enhance insulin synthesis, TCF7L2 significantly promotes the expression of the proinsulin genes *Ins1* and *Ins2*. Subsequently, TCF7L2 can upregulate PSCK1 and PSCK2 to cleave proinsulin into mature insulin and the C peptide (79, 80). Insulin secretion relies on islet β-cell exocytosis, which is controlled by the L-type Ca^2+^ channel CAV1.2. Upon membrane depolarization, Ca^2+^ influx through CAV1.2 triggers β-cell exocytosis (81–83). Ye et al. (83) found that TCF7L2 silencing reduced the expression of α2δ-1, an auxiliary subunit of CAV1.2, and alleviated the Ca^2+^ current, causing a voltage-insensitive response to high-glucose/depolarization-evoked stimulation, accompanied by insufficient insulin secretion. In addition, TCF7L2 can stimulate the PI3K/AKT pathway to promote insulin sensitivity and secretion, because TCF7L2 can bind to conserved TCAAAG motifs in the promoter of *PIK3R1* to upregulate PI3K (84). Li et al. found that TCF7L2 protein expression in the adipocytes, liver, and skeletal muscle is positively correlated with changes in homeostasis model assessment (HOMA)-IR (85), which indicates that TCF7L2 deficiency may induce IR. A recent study found that selective deletion of TCF7L2 in adipocytes leads to IR (63). In conclusion, accumulating evidence confirms that TCF7L2 plays a key role in metabolism and is responsible for maintaining β-cell function and insulin sensitivity. Under TCF7L2 dysfunction, T2D patients suffer more severe insulin deficiency and glucose metabolism disorders with a high risk of CVD (21, 36, 86).

### TCF7L2 Regulates Adipose Differentiation and Blood Lipid Homeostasis

Adipose tissue is the most abundant fat storehouse and exerts a chronic and profound influence on lipid metabolism and blood lipids. Blood lipid homeostasis is jointly maintained by adipose tissue and the cardiovascular system and is mutually beneficial (87, 88). A heat map of gene expression and gene set enrichment analysis showed alterations in several signaling pathways related to adipogenesis and metabolism in TCF7L2^−/−^ mice (63). Wnt signaling is well-documented to inhibit adipogenesis, in which the formation of the β-catenin/TCF7L2 complex is indispensable (89, 90). Inducible deletion of TCF7L2 promotes adipose differentiation and subcutaneous fat accumulation in TCF7L2^F/F^ mice (63).

**Figure 3:**
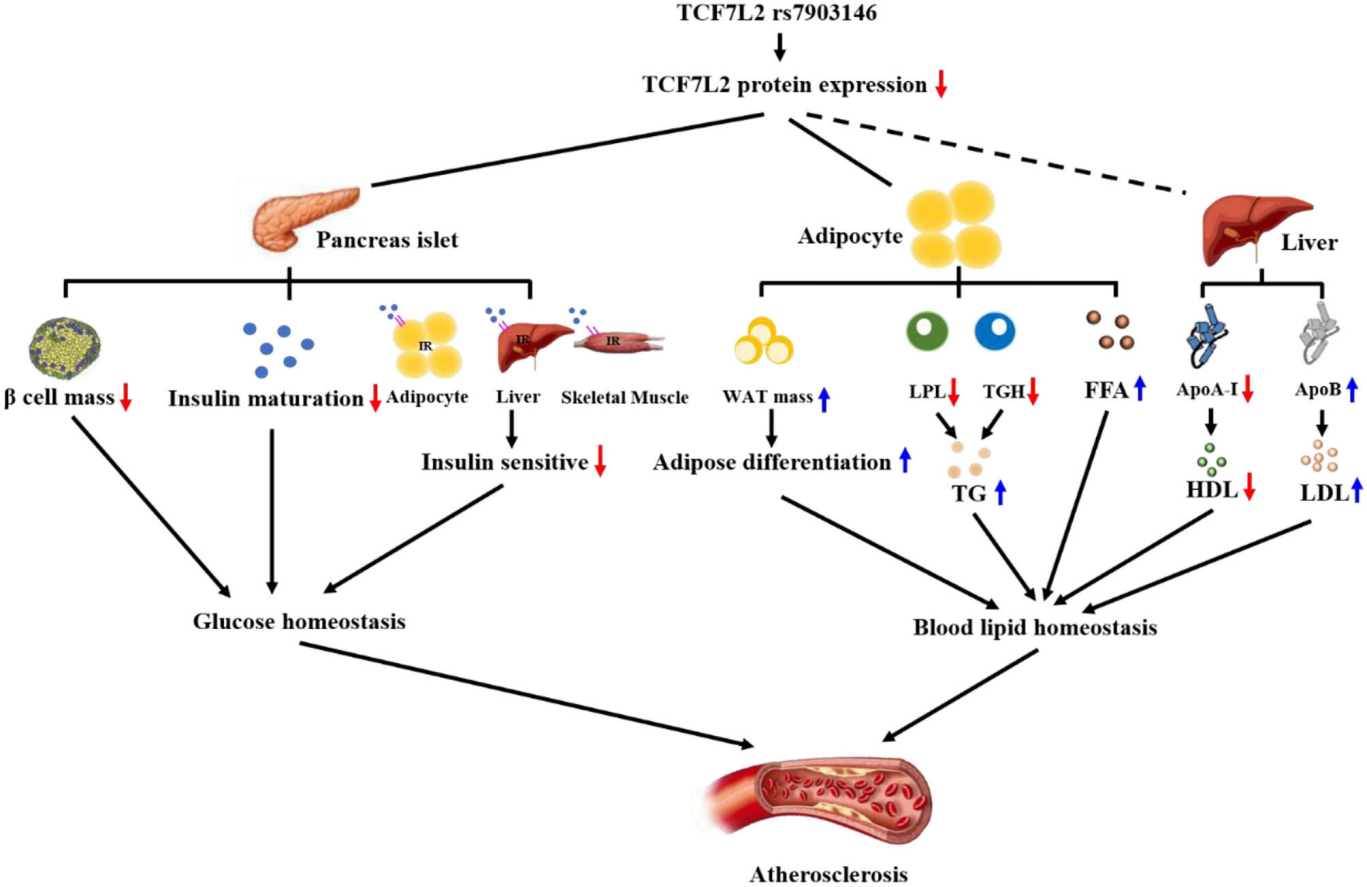
TCF7L2 rs7903146 is a link between metabolism disorders and atherosclerosis. TCF7L2 rs7903146 inhibits the expression of TCF7L2, thereby influencing glucose and blood lipid homeostasis. On the one hand, TCF7L2 rs7903146 induces hyperglycemia and insulin resistance by decreasing β-cell mass, proinsulin gene expression, Ca^2+^ voltage-gated channel activity, and insulin sensitivity of cells. On the other hand, TCF7L2 rs7903146 disrupts blood lipid homeostasis by increasing white adipocyte tissue (WAT) mass and harmful lipid components and decreasing HDL levels.

Atherogenic dyslipidemia is characterized by high TG, high fatty free acid (FFA), high LDL, and low HDL levels (91–94); particularly, high serum TG levels are very dangerous signals for myocardial infarction (95). TGs, which are small particles, can easily enter the arterial intima and induce subendothelial lipid accumulation and local inflammation (96). However, TCF7L2 expression can decrease serum TG levels with the help of lipoprotein lipase (LPL) and triglyceride hydrolases (TGH), both of which can hydrolyze TGs (22, 63). LPL is mainly anchored in the vascular endothelium and is sensitive to serum TG. Our previous study showed that TCF7L2 can bind to the promoter of *LPL* and promote LPL expression to decrease serum TG levels (97, 98). Another study showed using RNA seq analysis that the expression of TGH was decreased in TCF7L2^F/F^ mice, but the underlying mechanism remains unknown. Additionally, FFAs play a major role in inducing the endothelial prothrombotic state (99). A recent study showed that serum FFA concentrations increased dramatically after knocking out TCF7L2 in mice (72). In conclusion, TCF7L2 is a crucial regulatory element in metabolic balance. On the one hand, TCF7L2 regulates adipose differentiation *via* Wnt/β-catenin signaling and prevents adipocyte hypertrophy; on the other hand, TCF7L2 upregulates serum TG and FFA levels (Figure 3).

## Potential Anti-atherosclerotic Mechanism of TCF7L2

**Figure 4:**
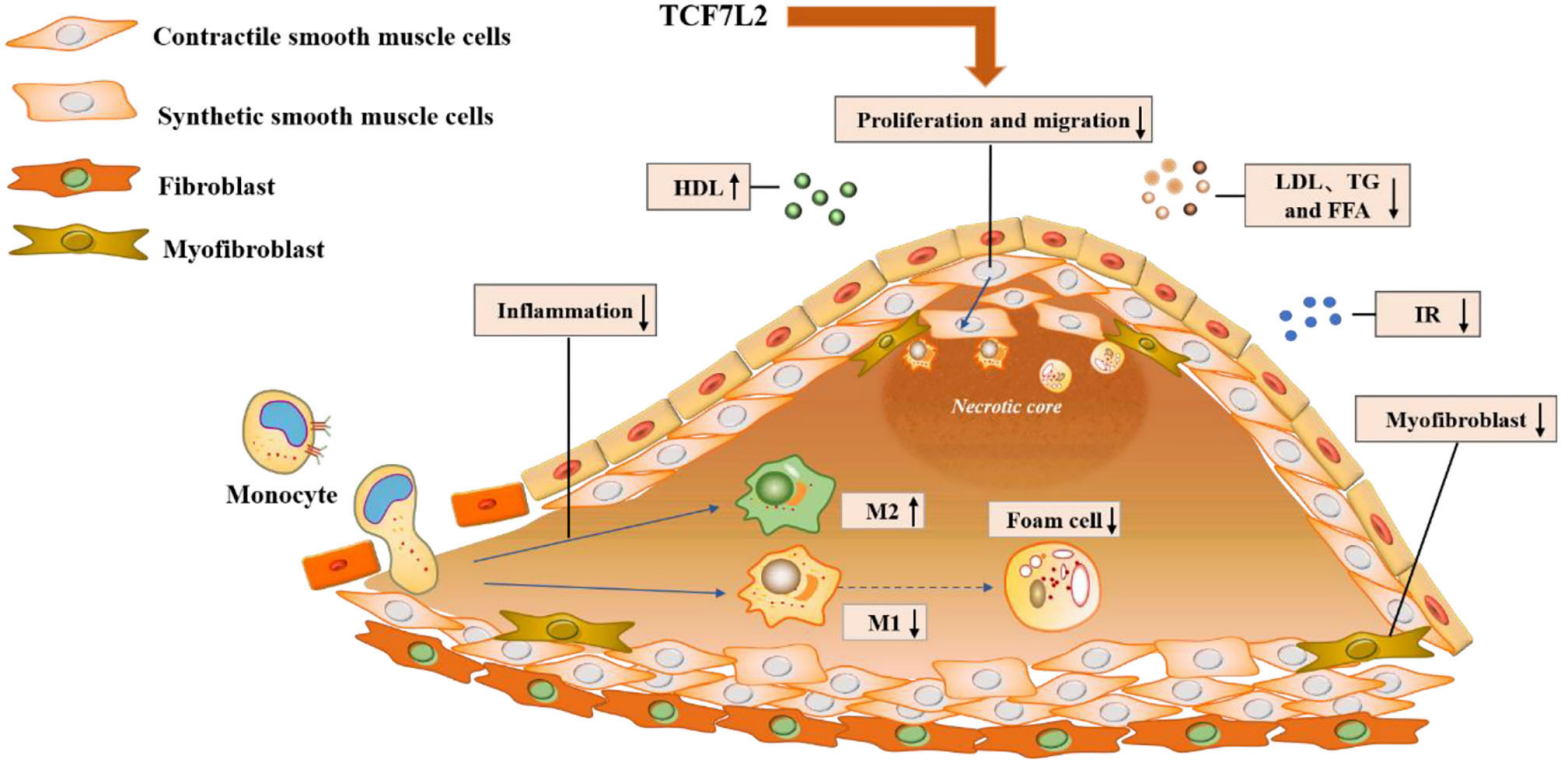
Molecular mechanisms underlying the action of TCF7L2 in anti-atherosclerosis. TCF7L2 exerts a protective role in atherosclerosis through multiple molecular mechanisms, including the inhibition of inflammation, VSMC proliferation and migration, dyslipidemia, insulin resistance, and foam cell and myofibroblast formation.

In addition to metabolic disorders, inflammatory infiltrates and neointimal hyperplasia aggravate the progression of atherosclerosis. Previously, we have illustrated TCF7L2 as a regulatory element in metabolism, but recent research has revealed other anti-atherosclerotic effects of TCF7L2, such as those against inflammation and neointimal hyperplasia (Figure 4).

### TCF7L2 Promotes M2 Polarization

Macrophages are the major inflammatory effectors in atherosclerotic lesions. After endothelial injury, monocytes are recruited by chemokines and differentiate into macrophages in the vascular wall. Macrophages are divided through the process of macrophage polarization into two primary phenotypes according to their roles in inflammation: pro-inflammatory M1 macrophages and anti-inflammatory M2 macrophages. Under IFN-γ or TNF-α stimulation, macrophages polarize into the M1 phenotype and intensify local inflammation. M1 macrophages secrete numerous pro-inflammatory factors, including ROS, IL-1, and TNF-α, in the atherosclerotic microenvironment where they can destroy the structure or function of blood vessels (100). Additionally, M1 macrophages are more likely to transform into foam cells because of excessive lipid intake. In contrast, M2 macrophages can delay the development of atherosclerosis by relieving local inflammation (101, 102). TCF7L2 partially regulates macrophage polarization (19, 103). *In vitro*, curcumin can upregulate TCF7L2 to suppress M1 macrophage-derived inflammation (103). Consistently, TCF7L2 can induce macrophage polarization toward the M2 phenotype by downregulating the lncRNA XIST (19). These results indicate that TCF7L2 may be an upstream factor that regulates macrophage polarization and exerts anti-inflammatory effects in the atherosclerotic microenvironment, but the underlying molecular mechanism needs further exploration.

### TCF7L2 Inhibits Neointimal Hyperplasia

During atherogenesis, VSMCs are characterized by two phenotypes: a contractile phenotype and a synthetic phenotype. VSMCs usually exhibit a contractile phenotype to maintain vascular elasticity. However, VSMCs transform from this contractile phenotype into a synthetic phenotype during atherogenesis and show strong proliferation and migration activities (104, 105). TCF7L2 has been proved to be negatively related to VSMC plasticity (20, 36). Mechanistically, TCF7L2 can directly bind to the promoter of GATA6 and upregulate the expression of SM-MHC, a well-established marker of the contractile VSMC phenotype. In addition, TCF7L2 can downregulate Sp1 expression to suppress the VSMC phenotypic switch (36, 106). Notably, Wnt/TCF7L2 dysfunction enhances Sp1/PDGF/JNK signaling and subsequently leads to extensive VSMC proliferation (107, 108). Taken together, TCF7L2 upregulates GATA6 expression while downregulating Sp1 expression to inhibit VSMC plasticity and neointimal hyperplasia. Understanding the mechanisms underlying the recruitment of VSMCs by TCF7L2 may provide novel insights into the pathogenesis of atherosclerosis.

Myofibroblasts play an active role in neointimal hyperplasia and have been reported in all stages of atherosclerotic CVD from plaque formation and rupture to restenosis after percutaneous coronary intervention (109). Transformation of fibroblasts into neointimal myofibroblasts causes collagen deposition and neointimal expansion under TGF-β stimulation (110–112). Meanwhile, myofibroblasts also secrete pro-inflammatory cytokines to aggravate atherosclerosis (113, 114). Contreras et al. found that TCF7L2 is highly expressed in fibroblasts, while it is barely expressed in myofibroblasts, and it behaves as a key control switch in the differentiation of fibroblasts to myofibroblasts (115, 116). During this process, TGF-β accelerates the degradation of TCF7L2 to induce myofibroblast formation *via* the ubiquitin-protease system (115).

## Therapeutic Strategies to Promote TCF7L2 Expression

TCF7L2 exerts multiple anti-atherosclerotic effects, including IR, lipid accumulation, and local inflammation. TCF7L2 is expected to be an alternative therapeutic target for atherosclerotic CVDs. Thus, the promotion of TCF7L2 expression is a more intuitive therapeutic strategy. Numerous studies have indicated that noncoding RNAs, including short noncoding RNAs (miRNAs) and long noncoding RNAs (lncRNAs), are master regulators of gene expression, and many are involved in the development of atherosclerosis (117). Serum miR-217 levels have been reported to be significantly increased in patients or mice with atherosclerosis (118). Mechanistically, miR-217 aggravates the progression of atherosclerosis by promoting endothelial dysfunction and inflammatory responses (119, 120). Yu et al. (121) showed that TCF7L2 is a downstream target of miR-217, as confirmed by dual luciferase reporter assays. Thus, suppression of miR-214 expression may be a possible treatment for atherosclerotic CVDs. Other miRNAs, such as miR-26b-5p, miR-17-5p, and miR-181-5p, show inhibitory effects on TCF7L2 expression similar to those of miR-217 (122, 123). Moreover, miR-26b-5p and miR-17-5p can promote adipogenic differentiation by downregulating TCF7L2 expression (124). MiR-17-5p can specifically bind to the 3′UTR of TCF7L2 mRNA and recruit the RNA-induced silencing complex (RISC) to degrade TCF7L2 mRNA (125). LncRNA also participates in the anti-atherosclerotic action of TCF7L2, wherein TCF7L2 downregulates the lncRNA XIST and promotes M2 polarization in THP-1 macrophages (19). Interestingly, TCF7L2 also participates in the protective effects of estradiol on the cardiovascular system (126). Tian et al. (126) generated a dominant-negative TCF7L2 mouse model *via* adenovirus transfection and found that overexpression of dominant-negative TCF7L2 significantly increased serum TG and hepatic lipid accumulation in male transgenic mice, but not in females; this finding caused the researchers to be curious about the relationship between TCF7L2 and estradiol. In the subsequent estradiol treatment, male transgenic mice showed an amelioration in serum TG and hepatic lipid accumulation, perhaps owing to the increased expression of TCF7L2 and the nuclear translocation of β-catenin. In general, TCF7L2 exerts a variety of anti-atherosclerotic effects by regulating downstream genes and is itself regulated by multiple controllable factors, making its use in clinical treatment applications potentially advantageous.

## Conclusions and Further Directions

TCF7L2, an important element in Wnt signaling, has attracted extensive attention since its discovery in 1998 (7). There are four functional structures in the TCF7L2 protein: the β catenin-binding domain, Groucho-binding sequence, HGM-DBD, and C-clamp (24, 25). HMG-DBD is responsible for identifying and binding to specific nucleotide sequences on the promoters of target genes to regulate their expression (17, 26). However, the activity of TCF7L2 is controlled by two contradictory regulatory elements: the co-activator β-catenin and co-repressor TLE/Groucho (28–30). TCF7L2 is mainly distributed in the nucleus and is usually occupied by TLE/Groucho, which suppresses the regulatory activity of TCF7L2 on target genes. However, activating the Wnt signaling pathway promotes the nuclear import of β-catenin, which competitively binds with TCF7L2 and exerts positive effects on TCF7L2. With the assistance of β-catenin, TCF7L2 participates in insulin secretion, adipogenesis, and maintenance of the blood lipid balance. During insulin secretion, TCF7L2 can increase proinsulin and PSCK1/PSCK2 to provide abundant insulin (79, 80). In addition, TCF7L2 upregulates the L-type Ca^2+^ channel CAV1.2, which promotes insulin efflux through exocytosis (81–83). TCF7L2-binding sites are present on the promoters of *LPL* and *TGH* genes, both of which are related to TG hydrolysis and can reduce serum TG levels (91–94).

In addition to metabolism, TCF7L2 can influence cell fate to alleviate atherosclerosis in the vascular endothelium. It is well established that local inflammation is an essential and forceful factor in the development of atherosclerosis, and macrophages are major participants in the inflammatory response (100–102). With different stimulations, macrophages can polarize into the pro-inflammatory M1 phenotype or anti-inflammatory M2 phenotype. TCF7L2 is involved in polarization of macrophages into the M2 phenotype, alleviating the local inflammatory response and atherogenesis (19, 103). Neointimal hyperplasia mainly occurs in the middle or late stages of atherosclerosis, especially after PCI (20, 36, 115, 116). During neointimal hyperplasia, VSMCs or fibroblasts proliferate and migrate to the vascular intima, where they transform into synthetic VSMCs or myofibroblasts, respectively, which synthesize and secrete large amounts of extracellular matrix. These changes eventually cause a decline in blood-vessel elasticity and lumen area, causing CVD patients to have more obvious clinical manifestations.

In terms of CVD prevention and treatment, we should pay attention to TCF7L2 rs7903146, which has been proven to be a causative SNP close to exon 4 (9, 46). Previous studies have shown that rs7903146 is closely associated with genetic susceptibility to T2D, and accumulating evidence indicates that rs7903146 increases CVD risk in the population, especially in subjects with diabetes (21, 127). Causally, rs7903146 alters the sequence of TCF7L2 mRNA, inserting an extra exon 4, and results in the reduction of TCF7L2 protein (47, 48). Owing to insufficient TCF7L2 protein, rs7903146 T-allele carriers are more likely to suffer from islet atrophy, dyslipidemia, and atherosclerosis, as shown in Figure 3. Thus, rs7903146 is a genetic biomarker to identify a population with a high risk of CVDs. Furthermore, TCF7L2 is a promising therapeutic target; we have provided a detailed description of its role in atherosclerosis from metabolism and inflammation to neointimal hyperplasia, which makes it suitable for clinical applications. However, a comprehensive understanding of TCF7L2 remains necessary to ensure that any future TCF7L2-based clinical treatment for CVD is safe and effective. Therefore, the following issues are worthy of further exploration: (i) the mechanism of TCF7L2 in independent Wnt signaling; (ii) factors affecting the conversion of TCF7L2 between co-repressors and co-activators; (iii) potential effects of TCF7L2 on FFA uptake in the intestine; (iv) mechanism underlying the induction of M2 polarization by TCF7L2; (v) potential inhibition of foam cells by TCF7L2; and (vi) potential regressive effect of TCF7L2 on vascular intimal hyperplasia. A better understanding of these issues will further reveal the molecular mechanism underlying the action of TCF7L2 in atherosclerosis, which is necessary for the development of new anti-atherosclerotic drugs in the future.

## Author Contributions

JYL, LZ, and PPH designed the writing framework. JYL and LZ wrote the original draft and drew figures. LZ, PPH, and XPOY modified and polished the manuscript. All authors contributed to the article and approved the submitted version.

## Funding

This work was supported by the Natural Science Foundation of China (No. 82170485), Natural Science Foundation of Hunan Province, China (No. 2019JJ40249), Outstanding Young Aid Program for Education Department of Human Province (No. 18B274), the Major Project of social science achievement review committee in Hunan Province (XSP20ZDI013), Key Project of Hunan Provincial Department of Education (20A427), and Graduate Research innovation Project of School of Nursing, University of South China (2021CX22).

## Conflict of Interest

The authors declare that the research was conducted in the absence of any commercial or financial relationships that could be construed as a potential conflict of interest.

## Publisher's Note

All claims expressed in this article are solely those of the authors and do not necessarily represent those of their affiliated organizations, or those of the publisher, the editors and the reviewers. Any product that may be evaluated in this article, or claim that may be made by its manufacturer, is not guaranteed or endorsed by the publisher.
